# 1-(4-Hy­droxy­phen­yl)-3-(3,4,5-tri­methoxy­phen­yl)thio­urea

**DOI:** 10.1107/S160053681004866X

**Published:** 2010-11-27

**Authors:** Hyeong Choi, Byung Hee Han, Yong Suk Shim, Sung Kwon Kang, Chang Keun Sung

**Affiliations:** a Department of Chemistry, Chungnam National University, Daejeon 305-764, Republic of Korea; b Department of Food Science and Technology, Chungnam National University, Daejeon 305-764, Republic of Korea

## Abstract

In the title compound, C_16_H_18_N_2_O_4_S, the dihedral angle between the hy­droxy­phenyl ring and the plane of the thio­urea moiety is 54.53 (8)°. The H atoms of the NH groups of thio­urea are positioned *anti* to each other. In the crystal, inter­molecular N—H⋯S, N—H⋯O, and O—H⋯S hydrogen bonds link the mol­ecules into a three-dimensional network.

## Related literature

For general background to tyrosinase, see: Ha *et al.* (2007 ▶); Kubo *et al.* (2000 ▶). For the development of tyrosinase inhibitors, see: Kojima *et al.* (1995 ▶); Cabanes *et al.* (1994 ▶); Casanola-Martin *et al.* (2006 ▶); Son *et al.* (2000 ▶); Iida *et al.* (1995 ▶). For thio­urea derivatives, see: Thanigaimalai *et al.* (2010 ▶); Klabunde *et al.* (1998 ▶); Criton (2006 ▶); Daniel (2006 ▶); Yi *et al.* (2009 ▶); Liu *et al.* (2009 ▶).
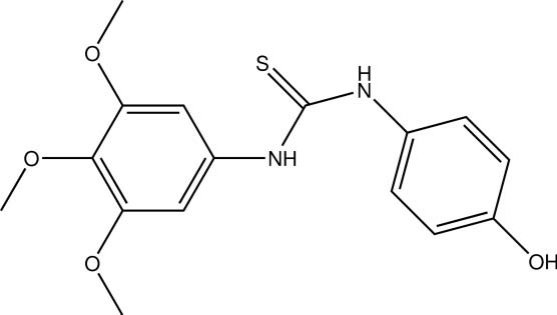


## Experimental

### 

#### Crystal data

C_16_H_18_N_2_O_4_S
*M* *_r_* = 334.38
Monoclinic, 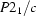

*a* = 10.5705 (5) Å
*b* = 12.8195 (7) Å
*c* = 12.4157 (7) Å
β = 99.434 (3)°
*V* = 1659.68 (15) Å^3^
*Z* = 4
Mo *K*α radiation
μ = 0.22 mm^−1^
*T* = 296 K
0.15 × 0.08 × 0.03 mm

#### Data collection

Bruker SMART CCD area-detector diffractometer
12193 measured reflections
3166 independent reflections
1723 reflections with *I* > 2σ(*I*)
*R*_int_ = 0.050

#### Refinement

*R*[*F* ^2^ > 2σ(*F* ^2^)] = 0.045
*wR*(*F* ^2^) = 0.121
*S* = 0.94
3166 reflections
219 parameters
H atoms treated by a mixture of independent and constrained refinement
Δρ_max_ = 0.50 e Å^−3^
Δρ_min_ = −0.27 e Å^−3^

### 

Data collection: *SMART* (Bruker, 2002 ▶); cell refinement: *SAINT* (Bruker, 2002 ▶); data reduction: *SAINT*; program(s) used to solve structure: *SHELXS97* (Sheldrick, 2008 ▶); program(s) used to refine structure: *SHELXL97* (Sheldrick, 2008 ▶); molecular graphics: *DIAMOND* (Brandenburg, 2010 ▶); software used to prepare material for publication: *WinGX* (Farrugia, 1999 ▶).

## Supplementary Material

Crystal structure: contains datablocks global, I. DOI: 10.1107/S160053681004866X/bt5417sup1.cif

Structure factors: contains datablocks I. DOI: 10.1107/S160053681004866X/bt5417Isup2.hkl

Additional supplementary materials:  crystallographic information; 3D view; checkCIF report

## Figures and Tables

**Table 1:** Hydrogen-bond geometry (Å, °)

*D*—H⋯*A*	*D*—H	H⋯*A*	*D*⋯*A*	*D*—H⋯*A*
N7—H7⋯S9^i^	0.81 (3)	2.61 (3)	3.383 (3)	160 (2)
N10—H10⋯O22^ii^	0.81 (2)	2.22 (3)	2.975 (3)	156 (2)
O17—H17⋯S9^iii^	0.97 (3)	2.25 (4)	3.211 (2)	173 (3)

Symmetry codes: (i) 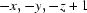; (ii) 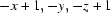; (iii) 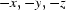.

**Fig. 1.**
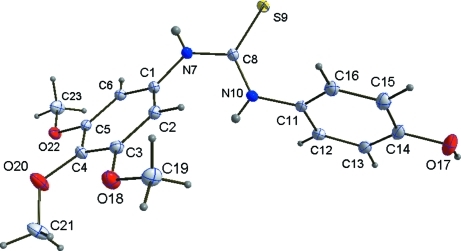
Molecular structure of (l), showing the atom-numbering scheme and 50% probability ellipsoids.

**Fig. 2.**
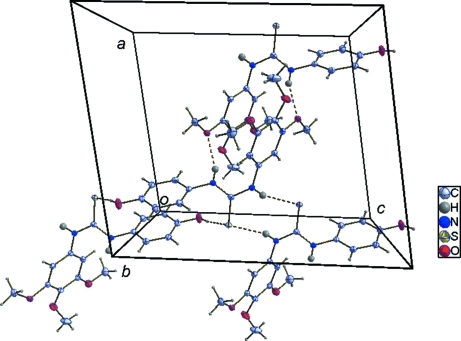
Part of the crystal structure of (I), showing 3-D network of molecules linked by intermolecular N—H···S, N—H···O, and O—H···S hydrogen bonds (dashed lines).

## supplementary crystallographic information

### Comment

Melanin production is primary responsible for the skin color, and melanin plays a key role in   protecting human skin from the harmful UV-induced skin damages. Tyrosinase is the key enzyme (Ha *et al.*, 2007; Kubo *et al.*, 2000)) that converts tyrosine to melanin and its inhibitors are the target molecules to develop and research anti-pigmentation agents for the application to skin care. Numerous potential tyrosinase inhibitors have been discovered from natural and synthetic sources, such as ascorbic acid (Kojima *et al.*, 1995), kojic acid (Cabanes *et al.*, 1994), arbutin (Casanola-Martin *et al.*, 2006) and tropolone (Son *et al.*, 2000; Iida *et al.*, 1995). Some thiourea derivatives, such as phenylthiourea (Thanigaimalai *et al.*, 2010; Klabunde *et al.*, 1998; Criton, 2006), alkylthiourea (Daniel, 2006), thiosemicarbazone (Yi *et al.*, 2009) and thiosemicarbazide (Liu *et al.*, 2009) have been also reported. During our works on developing potent whitening agents preventing the inadequacies of current whitening agents (poor skin penetration and toxicity) and minimizing the inhibitory effects of melanin creation, we have synthesized the title compound  from the reaction of 3,4,5-trimethoxyphenyl isothiocyanate and 4-aminophenol under ambient condition.

The 3,4,5-trimethoxyphenyl moiety is almost planar with r.m.s. deviation of 0.050 Å from the corresponding least-squares plane defined by the ten constituent atoms. The dihedral angle between the phenyl ring and the plane of thiourea moiety is 54.53 (8) °. In the crystal, intermolecular N—H···S, N—H···O, and O—H···S hydrogen bonds link the molecules into a three-dimensional network (Fig. 2, Table 1). The H atoms of the NH groups of thiourea are positioned *anti* to each other.

### Experimental

The 3,4,5-trimethoxyphenyl thiocyanate and 4-aminophenol were purchased from Sigma Chemical Co. Solvents used for organic synthesis were redistilled before use. All other chemicals and solvents were of analytical grade and were used without further purification. The title compound, (I), was prepared from the reaction of 3,4,5-trimethoxyphenyl isothiocyanate (0.20 g, 0.89 mmol) with 4-aminophenol (0.10 g, 1.10 mmol) in acetonitrile (6 ml). The reaction was completed within 30 min at room temperature. The reaction mixture was filtered rapidly and washed with n-hexane. Removal of the solvent gave a white solid (66% m.p. 499 K). Single crystals were obtained by slow evaporation of the ethanol at room temperature.

### Refinement

The H atoms of the NH and OH groups were located in a difference Fourier map and refined freely. The remaining H atoms were positioned geometrically and refined using a riding model with C—H = 0.93–0.96 Å, and with *U*_iso_(H) = 1.2*U*_eq_ (C) for aromatic and 1.5*U*_eq_(C) for methyl H atoms.

### Crystal data

**Table:**

C_16_H_18_N_2_O_4_S	*F*(000) = 704
*M**_r_* = 334.38	*D*_x_ = 1.338 Mg m^−^^3^
Monoclinic, *P*2_1_/*c*	Mo *K*α radiation, λ = 0.71073 Å
Hall symbol: -P 2ybc	Cell parameters from 2485 reflections
*a* = 10.5705 (5) Å	θ = 2.5–24.0°
*b* = 12.8195 (7) Å	µ = 0.22 mm^−^^1^
*c* = 12.4157 (7) Å	*T* = 296 K
β = 99.434 (3)°	Plate, colourless
*V* = 1659.68 (15) Å^3^	0.15 × 0.08 × 0.03 mm
*Z* = 4	

### Data collection

**Table:**

Bruker SMART CCD area-detector diffractometer	*R*_int_ = 0.050
φ and ω scans	θ_max_ = 26°, θ_min_ = 2.0°
12193 measured reflections	*h* = −13→8
3166 independent reflections	*k* = −15→8
1723 reflections with *I* > 2σ(*I*)	*l* = −12→15

### Refinement

**Table:**

Refinement on *F*^2^	0 restraints
Least-squares matrix: full	H atoms treated by a mixture of independent and constrained refinement
*R*[*F*^2^ > 2σ(*F*^2^)] = 0.045	*w* = 1/[σ^2^(*F*_o_^2^) + (0.0578*P*)^2^] where *P* = (*F*_o_^2^ + 2*F*_c_^2^)/3
*wR*(*F*^2^) = 0.121	(Δ/σ)_max_ < 0.001
*S* = 0.94	Δρ_max_ = 0.50 e Å^−^^3^
3166 reflections	Δρ_min_ = −0.27 e Å^−^^3^
219 parameters	

### Special details

**Table:**

Geometry. All e.s.d.'s (except the e.s.d. in the dihedral angle between two l.s. planes) are estimated using the full covariance matrix. The cell e.s.d.'s are taken into account individually in the estimation of e.s.d.'s in distances, angles and torsion angles; correlations between e.s.d.'s in cell parameters are only used when they are defined by crystal symmetry. An approximate (isotropic) treatment of cell e.s.d.'s is used for estimating e.s.d.'s involving l.s. planes.

### Fractional atomic coordinates and isotropic or equivalent isotropic displacement parameters (Å^2^)

**Table:**

	*x*	*y*	*z*	*U*_iso_*/*U*_eq_	
C1	0.2918 (2)	0.0884 (2)	0.50738 (19)	0.0393 (6)	
C2	0.3504 (2)	0.1779 (2)	0.4769 (2)	0.0418 (6)	
H2	0.3075	0.2214	0.4229	0.05*	
C3	0.4737 (2)	0.2018 (2)	0.5278 (2)	0.0433 (7)	
C4	0.5374 (2)	0.1378 (2)	0.6101 (2)	0.0460 (7)	
C5	0.4764 (2)	0.04838 (19)	0.63858 (19)	0.0373 (6)	
C6	0.3541 (2)	0.0237 (2)	0.5875 (2)	0.0397 (6)	
H6	0.314	−0.0364	0.6071	0.048*	
N7	0.1622 (2)	0.06459 (18)	0.4621 (2)	0.0459 (6)	
H7	0.119 (2)	0.045 (2)	0.507 (2)	0.055 (9)*	
C8	0.1061 (2)	0.06515 (19)	0.3571 (2)	0.0410 (6)	
S9	−0.05561 (6)	0.05352 (6)	0.32614 (6)	0.0539 (3)	
N10	0.1831 (2)	0.0730 (2)	0.28268 (18)	0.0489 (7)	
H10	0.259 (2)	0.0614 (19)	0.302 (2)	0.051 (8)*	
C11	0.1464 (2)	0.0882 (2)	0.1678 (2)	0.0420 (7)	
C12	0.1835 (2)	0.0159 (2)	0.0971 (2)	0.0486 (7)	
H12	0.2269	−0.0441	0.1241	0.058*	
C13	0.1559 (2)	0.0330 (2)	−0.0148 (2)	0.0492 (7)	
H13	0.1821	−0.015	−0.0629	0.059*	
C14	0.0900 (3)	0.1207 (2)	−0.0543 (2)	0.0505 (7)	
C15	0.0518 (3)	0.1923 (2)	0.0164 (2)	0.0548 (8)	
H15	0.006	0.2512	−0.0107	0.066*	
C16	0.0815 (2)	0.1766 (2)	0.1278 (2)	0.0501 (7)	
H16	0.0577	0.2259	0.1757	0.06*	
O17	0.0601 (2)	0.14204 (17)	−0.16395 (17)	0.0751 (7)	
H17	0.067 (3)	0.083 (3)	−0.211 (3)	0.113*	
O18	0.53853 (18)	0.28931 (15)	0.50440 (16)	0.0652 (6)	
C19	0.4781 (3)	0.3585 (2)	0.4229 (3)	0.0756 (10)	
H19A	0.5348	0.4155	0.4151	0.113*	
H19B	0.4006	0.3849	0.4437	0.113*	
H19C	0.458	0.322	0.3547	0.113*	
O20	0.6530 (2)	0.1626 (2)	0.67179 (19)	0.0943 (8)	
C21	0.7566 (3)	0.1977 (3)	0.6305 (4)	0.1032 (14)	
H21A	0.8255	0.2117	0.6892	0.155*	
H21B	0.7343	0.2606	0.5898	0.155*	
H21C	0.783	0.1457	0.5832	0.155*	
O22	0.54582 (15)	−0.01140 (14)	0.71848 (14)	0.0501 (5)	
C23	0.4836 (3)	−0.0992 (2)	0.7567 (2)	0.0669 (9)	
H23A	0.5424	−0.1348	0.8117	0.1*	
H23B	0.456	−0.1458	0.6969	0.1*	
H23C	0.4106	−0.0761	0.7871	0.1*	

### Atomic displacement parameters (Å^2^)

**Table:**

	*U*^11^	*U*^22^	*U*^33^	*U*^12^	*U*^13^	*U*^23^
C1	0.0324 (13)	0.0531 (16)	0.0315 (16)	−0.0018 (13)	0.0026 (12)	−0.0023 (13)
C2	0.0410 (15)	0.0485 (16)	0.0356 (16)	0.0011 (14)	0.0058 (13)	0.0035 (12)
C3	0.0436 (15)	0.0451 (16)	0.0428 (17)	−0.0060 (14)	0.0114 (14)	−0.0013 (14)
C4	0.0330 (14)	0.0615 (18)	0.0408 (17)	−0.0099 (14)	−0.0024 (13)	−0.0017 (14)
C5	0.0342 (13)	0.0489 (16)	0.0289 (15)	0.0021 (13)	0.0054 (12)	0.0002 (12)
C6	0.0350 (14)	0.0475 (15)	0.0368 (16)	−0.0040 (13)	0.0061 (12)	0.0015 (12)
N7	0.0326 (12)	0.0695 (17)	0.0347 (15)	−0.0071 (12)	0.0027 (12)	0.0069 (12)
C8	0.0348 (13)	0.0508 (16)	0.0358 (17)	−0.0001 (13)	0.0008 (13)	0.0021 (13)
S9	0.0326 (4)	0.0859 (6)	0.0421 (5)	−0.0061 (4)	0.0031 (3)	0.0022 (4)
N10	0.0282 (12)	0.0802 (18)	0.0378 (16)	0.0027 (13)	0.0036 (11)	0.0030 (12)
C11	0.0302 (13)	0.0629 (18)	0.0328 (17)	−0.0070 (13)	0.0050 (12)	0.0018 (14)
C12	0.0377 (15)	0.0613 (18)	0.0468 (19)	0.0016 (14)	0.0074 (14)	0.0023 (15)
C13	0.0494 (16)	0.0583 (18)	0.0414 (19)	−0.0069 (15)	0.0120 (14)	−0.0037 (14)
C14	0.0537 (17)	0.0601 (19)	0.0368 (18)	−0.0173 (16)	0.0043 (15)	0.0055 (15)
C15	0.0609 (18)	0.0531 (18)	0.050 (2)	−0.0024 (15)	0.0070 (16)	0.0067 (15)
C16	0.0483 (16)	0.0567 (18)	0.045 (2)	−0.0036 (15)	0.0070 (14)	−0.0031 (14)
O17	0.1081 (19)	0.0753 (15)	0.0396 (14)	−0.0098 (14)	0.0056 (13)	0.0103 (11)
O18	0.0618 (13)	0.0624 (13)	0.0684 (14)	−0.0216 (11)	0.0022 (11)	0.0138 (11)
C19	0.087 (2)	0.0554 (19)	0.086 (3)	−0.0068 (18)	0.020 (2)	0.0201 (19)
O20	0.0543 (14)	0.133 (2)	0.0873 (18)	−0.0382 (14)	−0.0146 (13)	0.0378 (15)
C21	0.049 (2)	0.092 (3)	0.163 (4)	−0.006 (2)	0.001 (2)	0.020 (3)
O22	0.0386 (10)	0.0654 (12)	0.0443 (12)	0.0019 (9)	0.0011 (9)	0.0128 (10)
C23	0.0617 (19)	0.078 (2)	0.060 (2)	0.0053 (18)	0.0080 (17)	0.0287 (17)

### Geometric parameters (Å, °)

**Table:**

C1—C6	1.377 (3)	C12—H12	0.93
C1—C2	1.386 (3)	C13—C14	1.371 (4)
C1—N7	1.426 (3)	C13—H13	0.93
C2—C3	1.387 (3)	C14—O17	1.374 (3)
C2—H2	0.93	C14—C15	1.376 (4)
C3—O18	1.371 (3)	C15—C16	1.381 (4)
C3—C4	1.394 (3)	C15—H15	0.93
C4—O20	1.370 (3)	C16—H16	0.93
C4—C5	1.389 (3)	O17—H17	0.97 (3)
C5—O22	1.368 (3)	O18—C19	1.416 (3)
C5—C6	1.380 (3)	C19—H19A	0.96
C6—H6	0.93	C19—H19B	0.96
N7—C8	1.341 (3)	C19—H19C	0.96
N7—H7	0.81 (3)	O20—C21	1.360 (4)
C8—N10	1.331 (3)	C21—H21A	0.96
C8—S9	1.696 (2)	C21—H21B	0.96
N10—C11	1.429 (3)	C21—H21C	0.96
N10—H10	0.81 (2)	O22—C23	1.423 (3)
C11—C16	1.375 (3)	C23—H23A	0.96
C11—C12	1.377 (3)	C23—H23B	0.96
C12—C13	1.389 (3)	C23—H23C	0.96
			
C6—C1—C2	120.9 (2)	C14—C13—H13	120.1
C6—C1—N7	118.1 (2)	C12—C13—H13	120.1
C2—C1—N7	120.9 (2)	C13—C14—O17	122.6 (3)
C1—C2—C3	119.1 (2)	C13—C14—C15	120.3 (3)
C1—C2—H2	120.4	O17—C14—C15	117.1 (3)
C3—C2—H2	120.4	C14—C15—C16	120.0 (3)
O18—C3—C2	123.3 (2)	C14—C15—H15	120
O18—C3—C4	116.0 (2)	C16—C15—H15	120
C2—C3—C4	120.6 (2)	C11—C16—C15	120.0 (3)
O20—C4—C5	117.2 (2)	C11—C16—H16	120
O20—C4—C3	123.5 (2)	C15—C16—H16	120
C5—C4—C3	118.9 (2)	C14—O17—H17	115 (2)
O22—C5—C6	123.7 (2)	C3—O18—C19	118.8 (2)
O22—C5—C4	115.5 (2)	O18—C19—H19A	109.5
C6—C5—C4	120.7 (2)	O18—C19—H19B	109.5
C1—C6—C5	119.7 (2)	H19A—C19—H19B	109.5
C1—C6—H6	120.2	O18—C19—H19C	109.5
C5—C6—H6	120.2	H19A—C19—H19C	109.5
C8—N7—C1	128.7 (2)	H19B—C19—H19C	109.5
C8—N7—H7	117.2 (19)	C21—O20—C4	124.5 (3)
C1—N7—H7	113.9 (18)	O20—C21—H21A	109.5
N10—C8—N7	116.9 (2)	O20—C21—H21B	109.5
N10—C8—S9	123.9 (2)	H21A—C21—H21B	109.5
N7—C8—S9	119.23 (19)	O20—C21—H21C	109.5
C8—N10—C11	127.3 (2)	H21A—C21—H21C	109.5
C8—N10—H10	117.7 (18)	H21B—C21—H21C	109.5
C11—N10—H10	114.6 (18)	C5—O22—C23	117.54 (19)
C16—C11—C12	120.1 (2)	O22—C23—H23A	109.5
C16—C11—N10	120.7 (2)	O22—C23—H23B	109.5
C12—C11—N10	119.1 (2)	H23A—C23—H23B	109.5
C11—C12—C13	119.8 (3)	O22—C23—H23C	109.5
C11—C12—H12	120.1	H23A—C23—H23C	109.5
C13—C12—H12	120.1	H23B—C23—H23C	109.5
C14—C13—C12	119.8 (3)		

### Hydrogen-bond geometry (Å, °)

**Table:**

*D*—H···*A*	*D*—H	H···*A*	*D*···*A*	*D*—H···*A*
N7—H7···S9^i^	0.81 (3)	2.61 (3)	3.383 (3)	160 (2)
N10—H10···O22^ii^	0.81 (2)	2.22 (3)	2.975 (3)	156 (2)
O17—H17···S9^iii^	0.97 (3)	2.25 (4)	3.211 (2)	173 (3)

Symmetry codes: (i) −*x*, −*y*, −*z*+1; (ii) −*x*+1, −*y*, −*z*+1; (iii) −*x*, −*y*, −*z*.
